# Revealing phosphorylation regulatory networks during embryogenesis of honey bee worker and drone (*Apis mellifera*)

**DOI:** 10.3389/fcell.2022.1006964

**Published:** 2022-09-26

**Authors:** Beibei Ma, Chuan Ma, Jianke Li, Yu Fang

**Affiliations:** Institute of Apicultural Research/Key Laboratory of Pollinating Insect Biology, Ministry of Agriculture, Chinese Academy of Agricultural Sciences, Beijing, China

**Keywords:** honeybee, drone, phosphorylation, worker, embryo

## Abstract

Protein phosphorylation is known to regulate a comprehensive scenario of critical cellular processes. However, phosphorylation-mediated regulatory networks in honey bee embryogenesis are mainly unknown. We identified 6342 phosphosites from 2438 phosphoproteins and predicted 168 kinases in the honey bee embryo. Generally, the worker and drone develop similar phosphoproteome architectures and major phosphorylation events during embryogenesis. In 24 h embryos, protein kinases A play vital roles in regulating cell proliferation and blastoderm formation. At 48–72 h, kinase subfamily dual-specificity tyrosine-regulated kinase, cyclin-dependent kinase (CDK), and induced pathways related to protein synthesis and morphogenesis suggest the centrality to enhance the germ layer development, organogenesis, and dorsal closure. Notably, workers and drones formulated distinct phosphoproteome signatures. For 24 h embryos, the highly phosphorylated serine/threonine-protein kinase minibrain, microtubule-associated serine/threonine-protein kinase 2 (MAST2), and phosphorylation of mitogen-activated protein kinase 3 (MAPK3) at Thr^564^ in workers, are likely to regulate the late onset of cell proliferation; in contrast, drone embryos enhanced the expression of CDK12, MAPK3, and MAST2 to promote the massive synthesis of proteins and cytoskeleton. In 48 h, the induced serine/threonine-protein kinase and CDK12 in worker embryos signify their roles in the construction of embryonic tissues and organs; however, the highly activated kinases CDK1, raf homolog serine/threonine-protein kinase, and MAST2 in drone embryos may drive the large-scale establishment of tissues and organs. In 72 h, the activated pathways and kinases associated with cell growth and tissue differentiation in worker embryos may promote the configuration of rudimentary organs. However, kinases implicated in cytoskeleton organization in drone embryos may drive the blastokinesis and dorsal closure. Our hitherto most comprehensive phosphoproteome offers a valuable resource for signaling research on phosphorylation dynamics in honey bee embryos.

## Introduction

Worker and drone bees are distinguishing castes in the honey bee colony. They are derived from fertilized (diploid) and unfertilized (haploid) eggs laid by queen bees (Nelson, 1915; Winston, 1987). Throughout the life cycle, worker and drone bees progress through well-defined stages with embryo, larva, pupa, and adult, undergoing a complete phenotypic metamorphosis (Nelson, 1915; Winston, 1987). Despite their similar life cycle, the worker and drone bees evolve significant differences in morphology, physiology, and social behaviors. Honey bee workers engage in broad repertoires of tasks for the good of colony survival, including hive cleaning, brood tending, larva nourishing, and foraging (Robinson, 1992, 2002). To achieve these biological roles, worker bees are equipped with well-developed glands, such as hypopharyngeal and mandibular glands to produce food for larvae, wax glands to secrete material for comb cell building, and scent glands to release pheromones or semiochemical compounds for communication with colony members (Nelson, 1915; Winston, 1987). On the other hand, drone bees perform a function that is quite limited but important to the colony, which is producing sperm and mating with virgin queens (Seidl., 1980). Accordingly, they exhibit anatomical and physiological adaptations for strong and forceful flying, and possess elaborate mating organs and powerful sense organs to pursue the virgin queen to mate in the open air (Nelson, 1915; Winston, 1987). Moreover, drone bees have developed a developmental cycle about 3 days longer (24 days) than worker bees (21 days) (Winston, 1987) and a body size about three times heavier than those of workers (Straus., 1911).

Embryogenesis is a complex rudimentary organ-generating process involving nuclear cleavage (<10 h), blastoderm formation (∼20 h), germ band differentiation (∼48 h), and dorsal closure (∼60 h), which occurs for about 72 h in both worker and drone bees (Fleig and Sander, 1986b). Due to the easily accessible conditions for embryonic development (34°C temperature, 80% relative humidity) (Nelson, 1915; Winston, 1987), the embryo is an ideal model used in honey bee genetic research (DuPraw, 1967). Several works have been reported to understand honey bee embryonic development from describing morphology to dissecting molecular underpinnings, such as detailed morphological differentiation (Fleig and Sander, 1986a; Woyke, 1998; Katzav-Gozansky et al., 2003), transcriptional modification (Pires et al., 2016; Netschitailo et al., 2022), and genome editing (Kohno et al., 2016; Hu et al., 2019; Nie et al., 2021; Wang et al., 2021; Değirmenci et al., 2022). Recently, our works on proteomics elucidated that embryos at different ages have tailored distinct proteome arsenals to underline the age-specific physiological demands of honey bees (Fang et al., 2014; Fang et al., 2015). However, knowledge of how protein phosphorylation modification regulates embryogenesis remains to be discovered.

The reversible phosphorylation of serine, threonine, and tyrosine residues is a critical regulatory mechanism that allows spatial and temporal regulation of the activation states and conformations of proteins, thereby regulating diverse downstream effects (Hunter, 1995; Raggiaschi et al., 2005). High-resolution mass spectrometry (MS) has become the platform for the confident identification of tens of thousands of phosphosites (p-sites) in other organisms (Olsen et al., 2006; Sharma et al., 2014). With current MS instrumentation, proteomics has identified phosphorylation signaling in honey bee brain (Bezabih et al., 2017) and hypopharyngeal glands (Qi et al., 2015), which play pivotal regulatory roles in the functionality of neurobiology and secretions of brood food (royal jelly) (Han et al., 2014; Qi et al., 2015). As such, phosphorylation is postulated to play an essential and widespread role in signaling during the development of honey bee embryos.

Phosphorylation contributes to the regulation of tissue configuration during the process of morphogenesis, during which the rudimentary organs are formed. Here, many phosphoproteins in the honey bee embryo may function as in *Drosophila*. For example, the phosphorylated ubiquitin carboxyl-terminal hydrolase is associated with compound eye formation and oocyte development (Bajpe et al., 2008; Shi et al., 2015), and the phosphorylated stromal interaction molecule homolog is involved in the construction of wing imaginal disc (Eid et al., 2008). Serine/threonine-protein kinase mig-15 is critical for cytoskeleton rearrangement, which is closely linked to the formation of the nervous system and the dorsal closure (Su et al., 1998; Shakir et al., 2006). Broad-complex core protein is directly related to the differentiation of photoreceptor cells in the compound eye and the formation of synapses (Brennan et al., 2001; Iyer et al., 2013). Phosphorylation plays a fundamental role in regulating morphogenesis during the embryogenesis of honey bees. However, phosphoproteins are mainly presented at low abundance and are overwhelmed by the excess of non-phosphorylated counterparts, which poses a technical challenge for the identification of phosphopeptides by MS. Enrichment of the low abundance phosphopeptides using different strategies, such as immobilized metal affinity chromatography (IMAC) and Titanium dioxide (TiO_2_), can yield significant expansion of phosphoproteome coverage. This is of paramount importance for phosphoproteome studies.

Although proteome drives the embryogenesis (Fang et al., 2014), and the mechanical discrepancy between the worker and drone has been illustrated so far (Fang et al., 2015), determining functionally relevant residues and establishing direct kinase-substrate relationships in the honey bee embryos is still elusive. Hence, the present study aims to portray time-resolved phosphorylation events that regulate the honey bee embryogenesis in both worker and drone bees, as well as the differences between these honey bee castes.

## Experimental procedures

### Chemical reagents

Unless the source is otherwise specified, all chemicals were purchased from Sigma-Aldrich (St. Louis., MO, United States). Titanium dioxide (TiO_2_, Titansphere, 5 μm particles) was bought from GL Science (GL-Sciences Inc., Japan). Ti^4+^-IMAC material was obtained from Dalian Institute of Chemical Physics, Chinese Academy of Sciences.

### Sample preparation

The honey bee (*Apis mellifera*) colonies used for sampling were raised at the Institute of Apicultural Research, Chinese Academy of Agricultural Sciences, Beijing, China. The worker and drone eggs from five colonies were sampled at 24, 48, and 72 h of age after being laid by the queens in worker or drone combs (for 3 h) based on our previous works (Fang et al., 2015). At each time point, 100 mg eggs (at least 1000 eggs) were collected from five colonies and stored at −80°C until further analysis. Three independent biological replicates were produced per time-point.

### Protein extraction and proteolytic digestion

Protein extraction was carried out according to our previously used method (Fang et al., 2014). Briefly, the egg samples were homogenized with a disposable pestle in lysis buffer, 8 M urea, 2 M thiourea, 4% 3-[(3-cholamidopropyl) dimethylammonio]-1-propane sulfonate (CHAPS), 20 mM Tris-base, 30 mM dithiothreitol (DTT), and phosphatase inhibitors (PhosSTOP, Roche, Basel, Switzerland) on ice. Afterward, the homogenates were centrifuged at 15,000× g for 15 min at 4°C to remove insoluble fractions. The supernatant was precipitated with ice-cold acetone at −20 °C for 30 min, followed by centrifuging twice at 15,000×g for 10 min at 4°C to pellet protein samples. Finally, the precipitate was extracted at room temperature (RT) for 10 min and dissolved in 40 mM NH_4_HCO_3_. Protein concentrations were determined by the Bradford assay.

To reduce the amount of denatured proteins and prevent the reformation of disulfide bonds, the samples were successfully exposed to DTT (final concentration 10 mM) and iodoacetamide (final concentration 50 mM). The sample was digested in solution according to the ratio 1:65 (enzyme: protein). After that, the digested samples were divided into two equal parts for phosphopeptide enrichment by TiO_2_ or Ti^4+^-IMAC.

### Phosphopeptide enrichment by titanium dioxide and Ti^4+^-immobilized metal affinity chromatography

To attain a deeper phosphoproteome coverage, the phosphopeptides in the worker and drone embryos were enriched by both TiO_2_ and Ti^4+^-IMAC. For phosphopeptide enrichment by TiO_2_ resin, the digested samples were reconstituted in 500 μL of the binding solution, 6.0% trifluoroacetic acid (TFA)/80% acetonitrile (ACN)/0.2 M dihydroxy-benzoic acid (DHB). A TiO_2_ slurry was prepared at a concentration of 10 mg/ml in a binding solution. About 50 μl of the prepared TiO_2_ slurry (10 mg/ml in binding solution) was added to the above mixture and incubated at RT for 60 min under vigorous shaking. Then, the supernatant was discarded, and the resin was washed sequentially with 200 μl of the binding solution, containing 0.5% TFA/50% ACN and 0.1% TFA/30% ACN. The phosphopeptides were eluted from the resin twice with 100 μl of a 0.5 mM K_2_HPO4. Each procedure of washing, and elution was rotated for 15 min at RT. The elutes were desalted by Zip-Tip C18 columns (Millipore, Billerica, MA, United States), vacuum dried, and stored at −80°C for further LC−MS/MS analysis.

To enrich phosphopeptides by Ti^4+^-IMAC, the immobilized Ti^4+^ polymer beads were prepared by overnight incubation of 10 mg in 100 mM Ti(SO_4_)_2_ solution at RT under gentle stirring. The Ti^4+^-IMAC beads were centrifuged at 20 000×g for 2 min to remove the supernatant, followed by washing with distilled water several times to remove the residual titanium ions. Finally, the obtained Ti^4+^-IMAC beads were dispersed in 30% ACN containing 0.1% TFA before usage. The phosphopeptide enrichment was carried out as described above in TiO_2_ enrichment.

### LC-MS/mass spectrometry measurement

The enriched phosphopeptides were resuspended in 0.1% FA and analyzed by an Easy-nLC 1000 (Thermo Fisher Scientific, Bremen, Germany; nano-electrospray ion source, spray voltage 2.3 kV, capillary temperature 275°C and S-Lens RF 55%) coupled to a Q-Exactive MS (Thermo Fisher Scientific, Bremen, Germany). The peptides were loaded onto a trap column (5.0 μm Aqua C18 beads, 2 cm long, 100 μm inner diameter fused silica, Thermo Fisher Scientific, Bremen, Germany) for 2 min in buffer A (0.1% formic acid) at a flow rate of 5 μL/min. The trap column effluent was transferred to a reversed-phase microcapillary column (15 cm long, 75 μm inner diameter fused silica trap column filing with 3.0 μm Aqua C18 beads, Thermo Fisher Scientific, Bremen, Germany). Reversed-phase separation of the peptides was done chromatographically with a 120-min gradient from 8% to 30% acetonitrile in 0.1% formic acid with a flow rate of 350 nl/min.

Mass spectra were retrieved by Xcalibur (version 2.2, Thermo Fisher Scientific, Bremen, Germany). The MS was run in positive ion mode, implementing dynamic exclusion. The MS continuously collected spectra in a data-dependent way over the entire gradient. MS1 precursor scan (m/z 350-1600) acquisition was performed in the Orbitrap using a nominal resolution of 70,000 at m/z 400. The top 20 highest intensity multiply charged precursor ions were fragmented by higher energy collisional dissociation (HCD), with normalized fragmentation energy of 35%. MS2 scans (m/z 200-2,000) were acquired in the Orbitrap mass analyzer using a resolution setting of 17,500 at m/z 400. The mass spectrometry proteomics data have been deposited to the ProteomeXchange Consortium (https://proteomecentral.proteomexchange.org) *via* the iProX partner repository (Ma et al., 2019) with the dataset identifier PXD033311.

### Phosphoprotein identification and site localization

For protein identification, the MS/MS spectra were searched using PEAKS search engine (version 8.5, Bioinformatics Solutions Inc., Waterloo, Canada) against the protein sequences of *Apis mellifera* (downloaded April 2022 from NCBI) and common contaminants, totaling 21,778 entries. The database search parameters were: precursor ion and MS/MS tolerances: 15 ppm and 0.05 Da; the enzyme specificity: trypsin; the maximum missed cleavages: 2; fixed modification: carbamidomethyl (C, +57.02 Da); and variable modification: oxidation (M, +15.99 Da), and phosphorylation (Ser, Thr, Tyr, +79.96 Da). The target and decoy sequence fusion strategy was used to control the false discovery rate (FDR) at 1% at the peptide level for protein identification. Scaffold PTM (Version 2.1.3, Proteome Software, Oregon, United States) was used to assess the localization probability of p-sites according to Ascore algorithm (Beausoleil et al., 2006). All MS/MS of phosphopeptides queries with an Ascore value for each site having at least 95% confidence were considered.

### Computational construction of site-specific kinase–substrate relations

To identify the kinase-substrate relations, GPS 5.0 software package (Group-based Prediction System, https://gps.biocuckoo.org) (Xue et al., 2008) was used to predict potential site-specific kinase–substrate relations (ssKSRs) in the worker and drone embryos systematically. Because the GPS 5.0 program doesn’t contain the PKs of *A.* propane sulfonate *mellifera*, here we first computationally identified 231 PKs for *A. mellifera* based on the HMM profiles and ortholog searches (Wang et al., 2014), whereas these PKs were classified in a hierarchical structure consisting of the group, family, and single PKs. Due to GPS can only predict the kinase-specific phosphosites at the PK cluster level, the links of 179 *A. mellifera* PKs with their corresponding GPS 5.0 predictors were manually formed. Then, we can predict the exact PKs of the experimentally identified phosphosites. We also retrieved the protein-protein interactions (PPIs) of *A. mellifera* from the database of STRING (Szklarczyk et al., 2015) v10 (https://www.string-db.org/). Then by BLAST search, the proteins of PPIs were mapped to the benchmark sequences of the proteome in *Apis mellifera* L. downloaded from the database of Bee Base (www.beebase.org, version 3.2) (Elsik et al., 2016). Finally, 906,294 non-redundant PPIs of 8,336 proteins for *Apis mellifera* L. were obtained. Furthermore, we adopted the PPIs as a filter to reduce the potential false positive hits of the predicted ssKSRs (Song et al., 2012). The GPS predictions were reserved only if the kinase–substrate relations were supported by the data of PPIs (Song et al., 2012). Then the predicted ssKSRs were employed to construct the kinase-substrate phosphorylation networks (KSPNs), which were visualized with Cytoscape (version 3.4.0). Again, based on the hypothesis that kinases with higher activity may phosphorylate on more sites (Song et al., 2012), the number of ssKSRs for a kinase was used as the number representing the activity of a kinase.

### Quantification of phosphoprotein abundance levels

To evaluate the relative abundance level of phosphoproteins in the honey bee worker and drone embryos, raw MS data were analyzed by PEAKS Q module (version 8.0, Bioinformatics Solutions Inc., Waterloo, Canada). One sample was automatically selected as a reference. Based on an expectation-maximization algorithm, feature detection was employed separately on each sample. A high-performance retention time algorithm was used to align the features of the same peptide from different samples (Lin et al., 2013). Peptide ion abundance in the three replicates was subjected to calculate the expression level of each protein. The abundance level of a protein was quantified with the sum of the ion peak intensity of the three most abundant precursors of the tryptic peptides. Calculations of the protein *p*-value (one-way ANOVA) were made on the sum of the normalized abundances across all runs (Lin et al., 2013). Proteins were significantly changed in abundance levels only when they attained the criteria of *p*-values < 0.05 and a fold change ≥1.5.

### Bioinformatics analysis

To understand the biological function of the identified phosphoproteins involved in the embryogenesis of the honey bees, Gene Ontology (GO) term was enriched by ClueGO v2.3.3 (http://www.ici.upmc.fr/cluego/) (Bindea et al., 2009), a Cytoscape plugin. The significantly enriched functional GO categories in biological processes were reported by comparing the input data with the background of GO annotations in the honey bee genome, applying a right-sided hyper-geometric test. The nodes in functionally grouped networks were linked based on kappa score (0.4). Functional categories were only considered to be significantly enriched when the *p*-value was <0.05. An FDR was controlled with a Bonferroni step-down test to correct the *p*-value of GO terms. Moreover, the identified phosphoproteins were assigned into specific functional categories according to the annotations of the proteins.

To gain insight into the statistically significant biological pathways of the identified phosphoproteins, KEGG Orthology-Based Annotation System (KOBAS, http://kobas.cbi.pku.edu.cn) (Xie et al., 2011) was applied as previously described in protocol (Fang et al., 2014).

### Western blotting analysis

To validate two p-sites in different developmental phases of worker and drone embryos, Western Blotting analysis was done as previously described (Yu et al., 2010) using the ECL (enhanced chemiluminescence). The primary rabbit polyclonal antibodies (Abcam, MA, United States) were anti- Cyclin-dependent kinase 1 Tyr^15^ (CDK1 Y15) and anti-Mitogen activated protein kinase 3 Thr^564^ (MAPK3 T564), at dilutions of 1:4000 and 1:3000, respectively. The secondary antibody (Ptm-bio, Hangzhou, China) was horseradish peroxidase-conjugated goat anti-rabbit at a dilution of 1:5000. Samples of 10 μg protein were separated by stacking (4%) and separating (12%) SDS-PAGE gels, and each sample was run in triplicates. Protein was transferred to nitrocellulose membranes. The protein bands were visualized by chemiluminescence and quantified by densitometry using a Quantity-one image analysis system (Bio-Rad, Hercules, CA, United States). The protein abundance was normalized by GAPDH. The student t-test was used for statistical analysis of protein abundance.

### Activity assay of CDK5, MAPK3, and CDK1

The activity of the crucial kinases, CDK5, MAPK3, and CDK1, were measured according to the instruction. The kinase assay kits were bought from MyBiosource, Inc. (San Diego, CA, United States; Cat. No. MBS2882387, MBS2882543 for the activity assay kits of CDK5, MAPK3, and CDK1, respectively). Briefly, honey bee embryos were homogenized in ice-cold PBS with a glass homogenizer. The resulting suspension was subjected to ultra-sonication to break the cell membranes further. Then the homogenates were centrifuged for 15 min at 1500 × g. Remove the supernatant and assay immediately. The assay samples and standards were incubated with kinase-HRP conjugate in a pre-coated microtiter plate for 1 hour. After the incubation period, the wells were decanted and washed five times. The wells were then incubated with a substrate for HRP enzyme. The product of the enzyme-substrate reaction forms a blue-colored complex. Finally, a solution was added to stop the reaction, turning the solution yellow. The optical density of each plate was measured with Multiskan GO (Thermo Fisher Scientific, Waltham, MA, United States) at a wavelength of 450 nm. The standard curve is used to determine the activities of samples.

### RNA interference

To validate the role of the crucial kinase in the phosphorated network, the CAMK2 was selected for RNA interference. The siRNA sequences of CAMK2 were designed according to the technical requirements and then synthesized (BGI, BJ, China; sequences were in Supplementary Table S1). The injections of siRNA were performed based on the previously described method (Fang et al., 2015). Briefly, each freshly laid embryo (within 3 h after oviposition) was injected into the dorsal posterior side with 5 nl siRNA solution (concentration at 2.5 µg/ul). In contrast, the equivalent nonsense siRNA solution and sterile water were injected as controls. The injection was conducted under a stereoscopic microscope (CSOIF XTZ3) using an injector (Eppendorf Femtojet). The injection time was 0.1 s, and the pressures of injection and balance were 800 and 60 hPa, respectively. The injected embryos were immediately placed in an incubator at 34°C with a relative humidity of 80% until hatched. Then the larvae were transferred into 48-well plates and reared according to the described protocol (Kaftanoglu et al., 2010). The survival larvae were collected at 2d embryo, 2d, and 5d larvae for the following Western-Blotting and quantitative PCR (qPCR) analyses.

### Real-time qPCR

To investigate the transcription activity of CAMK2 after RNAi, Real-time qPCR was carried on. Total RNA extracted from the embryo or larva samples harvested after RNAi (TRIzol reagent, Invitrogen, United States) was used to generate cDNA using Reverse Transcriptase kit reagents (Transgen, China) following the manufacturer’s instructions. The primer pairs are designed and synthesized by BGI (Supplementary Table S1), and glyceraldehyde-phosphate dehydrogenase (GAPDH) was used as a reference gene to normalize data. Briefly, cDNA was quantified in duplicate for each sample using LightCycler 480 SYBR Green on a LightCycler 480. Reaction parameters were: 15 min at 95°C, 45 cycles of (15 s at 94°C, 25 s at 55°C, 20 s at 72°C). Data is analyzed as fold change above proliferative condition mRNA level using 2^-△△Ct^ values. The statistical analysis was performed by one-way ANOVA (SPSS version 16.0, SPSS, lnc., IL, United States).

## Results

Three-time points representing significant stages of embryonic development were selected: division of the embryo cell and formation of the blastoderm (0–26 h), shaping of the germ band and germ layer (26–46 h), and organogenesis (46–72 h). Pearson correlation coefficients between replicates of the same samples were above 0.91 (Supplementary Figure S1), regardless of TiO_2_ and Ti^4+^-IMAC enrichment, showing excellent technical and biological reproducibility.

### Time-resolved phosphoproteome of worker embryos

In worker embryos, 1014 and 1994 p-sites were localized in phosphopeptides enriched by TiO_2_ and Ti^4+^-IMAC, of which 734 p-sites overlapped (Supplementary Figure S2). In total, 2386 p-sites from 5637 phosphopeptides in 2145 phosphoproteins were identified. The abundance of phosphoserine (pS), phosphothreonine (pT), and phosphotyrosine (pY) were around 85%, 14%, and 1%, respectively. A similar distribution of p-sites was observed in phosphopeptides enriched by the TiO_2_ and Ti^4+^-IMAC methods. The details of identified proteins, peptides, and p-sites are in Supplementary Tables S2–S8.

During the embryogenesis of workers, the identified proteins were significantly enriched in 4 function groups: regulation of catalytic activity, protein phosphorylation, establishment or maintenance of cell polarity, and cytoskeleton organization, and were assigned into 16 functional categories according to annotation (Supplementary Figure S3; Supplementary Table S9). The major represented categories were transcription (14.3%), followed by morphogenesis (13.9%), cell cycle control/apoptosis (13.0%), cytoskeleton (10.8%), and signal transduction (9.2%). Notably, in most categories, the proteins were overrepresented at 72 h, compared to at 24 and 48 h.

**FIGURE 1:**
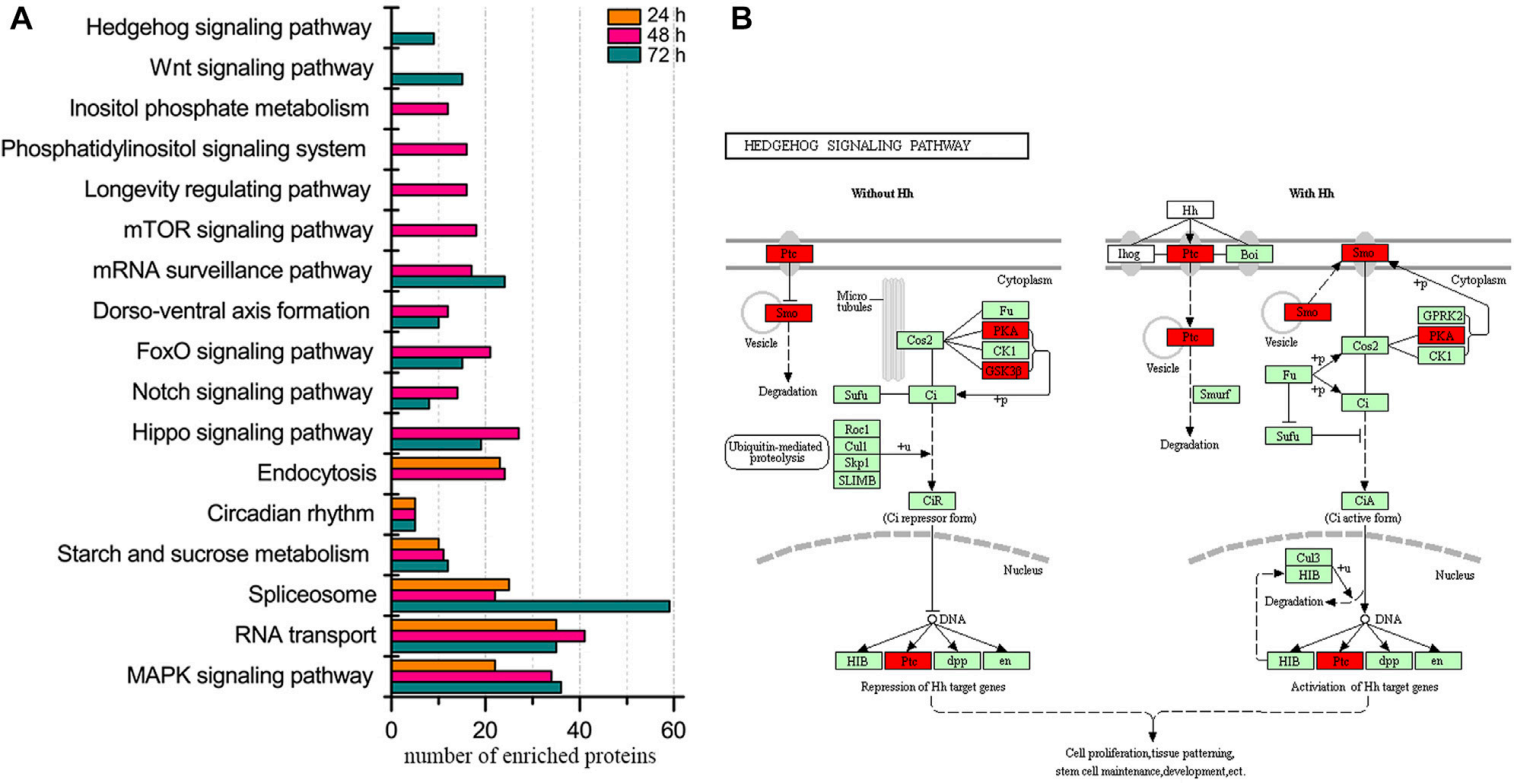
Biological pathway enrichment of identified proteins in the embryos of honey bee workers. **(A)** Comparison of enriched biological pathways of the identified proteins in the embryo of the honey bee workers aged 24, 48, and 72 h. Significantly enriched pathways are analyzed by KEGG Orthology-Based Annotation System (KOBAS, https://kobas.cbi.pku.edu.cn). A hypergeometric statistic test conducts the pathway enrichment. The Benjamini and Hochberg FDR correction is used to correct the probability values, and only the corrected *p* < 0.05 is considered statistically significant enriched pathways. **(B)** Representative enriched pathway map from KOBAS. Green labeled boxes are the protein references of honey bees annotated to the KEGG PATHWAY database. Highlighted red ones indicate the protein entries mapped to the significantly enriched pathway.

Regarding the enriched biological pathways, MAPK signaling pathway, RNA transport, spliceosome, starch and sucrose metabolism, and circadian rhythm were present at all three ages of embryos (Figure 1, Supplementary Table S10). Moreover, the pathway of mTOR signaling, longevity regulation, phosphatidylinositol signaling system, and inositol phosphate metabolism were specifically enriched in 48 h embryos. Wnt and hedgehog signaling pathways were uniquely enriched in 72 h embryos, and no exclusive pathway was enriched in embryos of 24 h.

**FIGURE 2:**
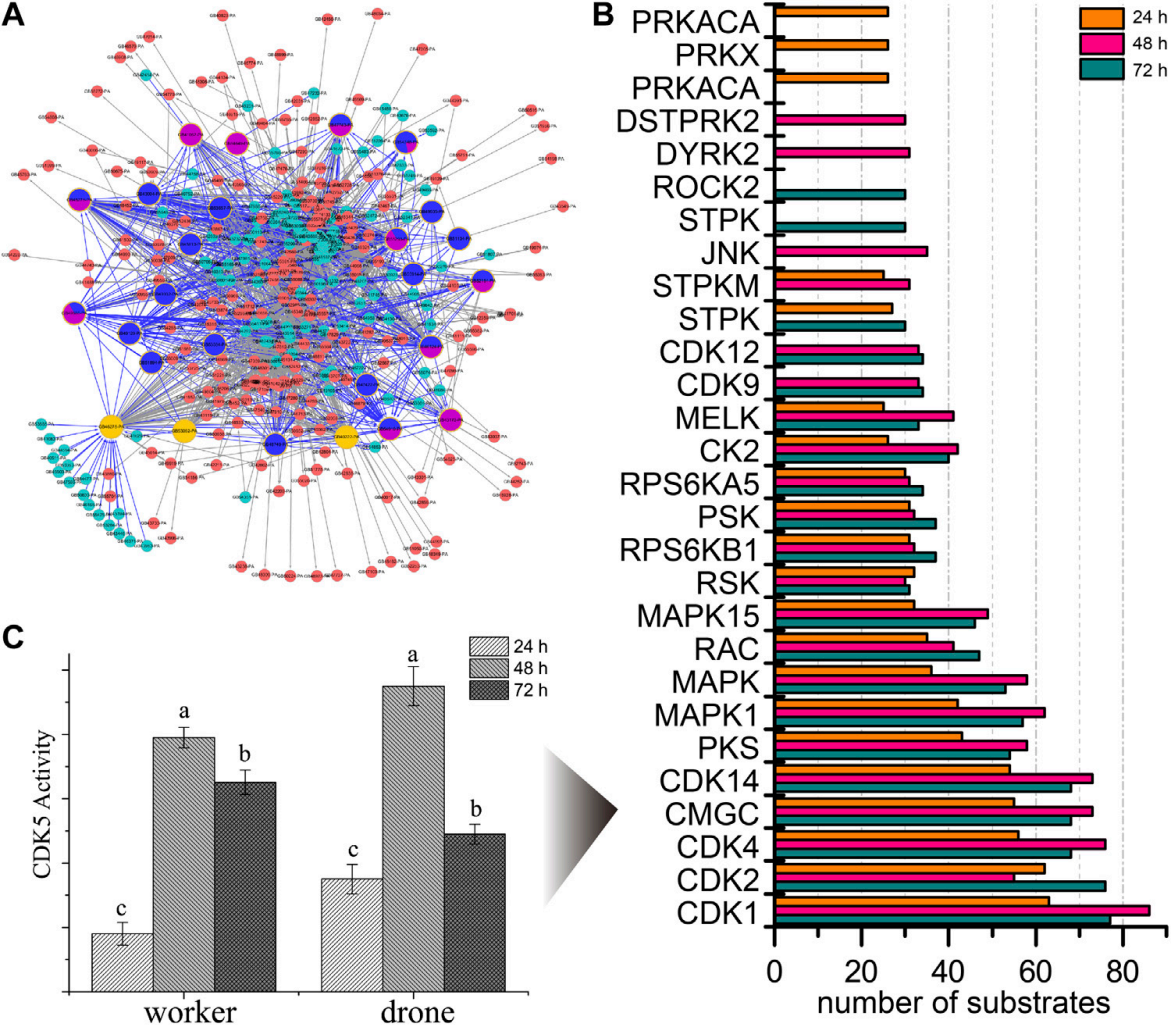
The predicted site-specific kinase-substrate relations (ssKSRs) in worker embryos. GPS 2.1 software package (Group-based Prediction System, https://igps.biocuckoo.org) was used to predict potential ssKSRs systematically. Protein-protein interactions of *A. mellifera* retrieved from the database of STRING v10 (https://www.string-db.org/) were used to reduce possible false positive hits of the predicted ssKSRs. **(A)** The network was constructed from the identified phosphoproteins in 24 h worker embryo. The highlighted proteins are kinases identified in this study. **(B)** The kinase activity analyses for the phosphoproteome of the worker. The top 20 high-activity kinases over the three stages of development are compared. **(C)** The enzymatic activity of CDK5 in different phases during the embryogenesis of worker and drone. Error bar is the standard deviation. “a” is significantly higher than “b” and “c”, “b” is significantly higher than “c”.

To explore the kinases involved in embryogenesis, 166 kinases were predicted, and their KSPNs were constructed (Figure 2A, Supplementary Figure S4; Supplementary Tables S11–S13). Of the top 20 high-activity kinases over the three stages of development, 16 were represented in all stages of worker embryogenesis (Figure 2B). Moreover, three kinases (PRKACA, PRKX, and PRKAC), two kinases (DSTPRK2 and Dyrk2), and ROCK2 & STPK were uniquely observed in 24, 48, and 72 h embryos, respectively.

To further study the phosphorylation events in kinases, the occupancy of p-sites in the high-activity kinases was investigated. The number of pS in MAST3 (5, 5, and 7 at 24, 48, and 72 h), CDK12 (1, 14, and 17 at 24, 48, and 72 h) and MARK2 (1, 1, and 2 at 24, 48, and 72 h) was increased during embryogenesis; the number of pT in CDK2 (0, 0, and 1 at 24, 48 and 72 h), CDK1 (0, 0, and 1 at 24, 48 and 72 h), mig-15 (0, 0, and 1 at 24, 48 and 72 h) and CDK12 (0, 1, and 1 at 24, 48 and 72 h) followed the same trend; pY in CDK2 (1, 2, and 1 at 24, 48, and 72 h) and CDK1 (1, 2, and 1 at 24, 48, and 72 h) were the significant p-sites in 48 h embryos (Supplementary Table S14).

**FIGURE 3:**
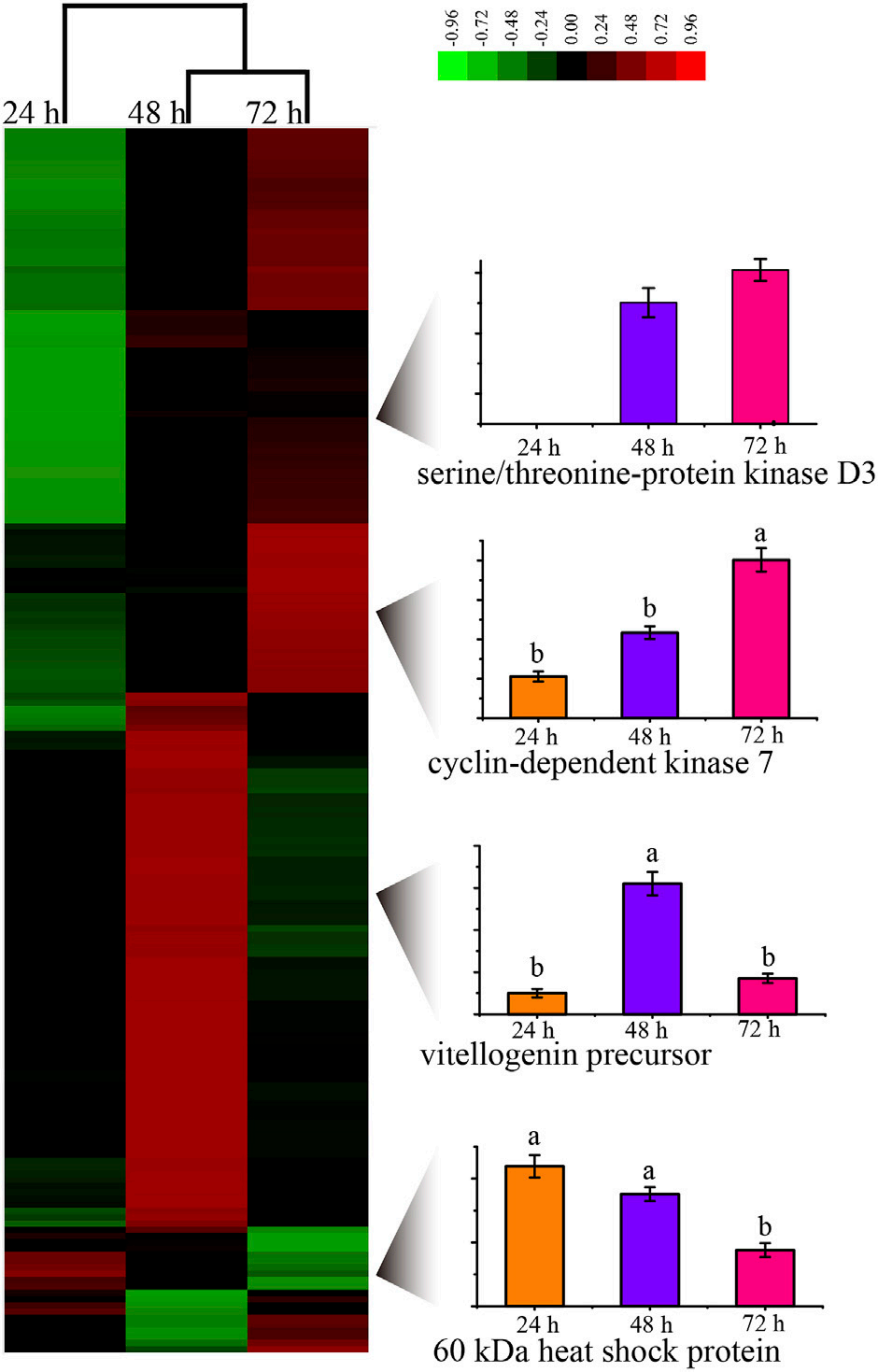
Unsupervised hierarchical clustering of the differentially expressed (fold change≥1.5 and *p* < 0.05) proteins in the embryo of honey bee workers. The columns represent the embryonic age (24, 48, and 72 h), and the rows represent the individual proteins. The up- or downregulated proteins are indicated by red and green color codes, respectively. The color intensity changes with the protein expression level, as noted on the key bar on the top right. The histograms denote the expression trend of the representative proteins during the embryogenesis of the honey bee workers aged 24, 48, and 72 h, respectively.

By evaluating the phosphoproteome profile change during the development of the worker embryo, we found 195 phosphoproteins differentially regulated in a total of 2145 phosphoproteins (Figure 3, Supplementary Table S15). Among these, 10, 95, and 90 proteins were up-regulated in 24, 48, and 72 h embryos, respectively. The up-regulated phosphoproteins at 48 h were mainly enriched in the regulation of protein metabolic process and the negative regulation of gene expression; the up-regulated proteins at 72 h were enriched in the positive regulation of molecular function and the spliceosome pathway.

### Time-resolved phosphoproteome of drone embryos

In the drone embryo, 636 and 882 p-sites were assigned in the phosphopeptides enriched by TiO_2_ and Ti^4+^-IMAC, respectively (Supplementary Figure S5). In total, 1166 p-sites from 2928 phosphopeptides in 1423 phosphoproteins were identified. The percentages of pS, pT, and pY were around 87%, 12%, and 1%, respectively. The identified proteins, peptides, and p-sites are in Supplementary Tables S13–S22.

According to the functional annotation, phosphoproteins were assigned into 16 functional categories (Supplementary Figure S6). The major represented categories were proteins related to morphogenesis (16.7%), followed by proteins associated with cell cycle control/apoptosis (13.1%), transcription (12.5%), signal transduction (10.5%), and cytoskeleton (10.4%). Intriguingly, most categories of proteins expressed were overrepresented at 48 h, compared to the two other time points.

During embryogenesis of the drone bees, the enriched pathways shared over the three ages were: MAPK signaling pathway, RNA transport, and circadian rhythm (Supplementary Figure S7; Supplementary Table S10). Moreover, mTOR signaling pathway, spliceosome, and dorso-ventral axis formation were specifically enriched in 72 h embryos. No pathways were exclusively enriched in the 24 and 48 h embryos.

At the same time, 162 kinases were predicted, and the KSPNs were constructed in drone embryogenesis. Of the top 20 high-activity kinases present during the three ages of growth, 14 kinases were present in the whole process of embryogenesis (Supplementary Figure S8, Supplementary Tables S23–S25). Moreover, five kinases (PKG, MELK, PRKACA, PRKX, and PRKAC) and two kinases (JNK and MYO3B) were specifically involved in 24 and 72 h embryos.

Furthermore, the occupancy of p-sites in the high-activity kinases was also observed. pS in STPK (1, 2, and 1 at 24, 48, and 72 h), CDK12 (12, 13, and 12 at 24, 48, and 72 h), and AbI (2, 9, and 5 at 24, 48, and 72 h) was the most mode of p-sites in the 48 h embryos. The number of pT increased along with the embryonic development in kinases STPK (1, 2, and 4 at 24, 48, and 72 h) and CDK7 (0, 1, and 1 at 24, 48, and 72 h) (Supplementary Table S26).

Amongst 1423 phosphoproteins, 71 were changed in their expression level during the embryogenesis of honey bee drone (Supplementary Figure S9; Supplementary Table S27), of which 20, 33, and 18 were upregulated in the three developmental stages, respectively. The up-regulated proteins in 24 h embryos were significantly enriched in GO terms involved in ATP hydrolysis coupled ion transmembrane transport. No GO term was enriched in the two other embryonic phases.

### Phosphoproteomic difference between the worker and drone embryos at 24 h

**FIGURE 4:**
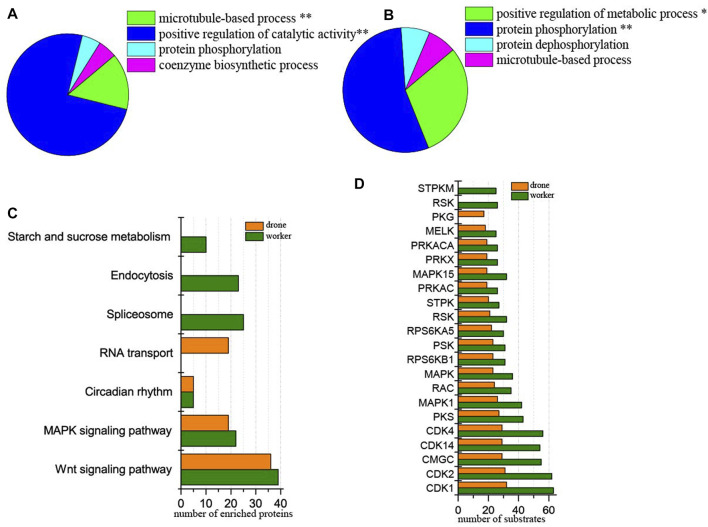
Comparison of the phosphoproteins identified in worker and drone embryo at 24 h. **(A)** ClueGO analysis of the phosphoproteins identified in 24 h worker embryos. The significantly enriched functional GO categories in biological processes were reported by comparing the input data with the background of GO annotations in the honey bee genome, applying a right-sided hyper-geometric test. The nodes in functionally grouped networks were linked based on kappa score (0.4). An FDR was controlled with a Bonferroni step-down test to correct the *p*-value of GO terms. The single and double asterisks indicate significant enrichment at *p* < 0.05 and *p* < 0.01statistical levels, respectively. **(B)** ClueGO analysis of the phosphoproteins identified in 24 h drone embryos. **(C)** Comparison of the biological pathways enriched in worker and drone embryo at 24 h. **(D)** Comparison of the kinase activity between the embryogenesis of worker and drone at 24 h.

At 24 h of age, 3114 p-sites from 3015 phosphopeptides in 1254 phosphoproteins were identified (2617 p-sites from 2451 phosphopeptides in 1061 phosphoproteins of worker embryos and 1646 p-sites from 1449 phosphopeptides in 708 phosphoproteins of drone embryos). In worker embryos, the proteins were enriched in the functional groups: positive regulation of catalytic activity and microtubule-based process (Figure 4A). In drone embryos, protein phosphorylation and positive regulation of metabolic processes were enriched (Figure 4B).

In comparing differences in enriched biological pathways between the embryos of worker and drone honey bees, in 24 h embryos, the pathways of wnt signaling and MAPK signaling was commonly enriched in worker and drone. In worker embryos, spliceosome, endocytosis, and starch and sucrose metabolism were uniquely enriched, whereas, in drone embryos, RNA transport pathways were exclusively enriched (Figure 4C, Supplementary Table S10).

Furthermore, the activity of the critical kinases in the worker and drone embryos was compared. At 24 h, the activity of most kinases was higher in worker embryos, whereas only kinase PKG was high in drone embryos (Figure 4D).

In comparing site occupancy between worker and drone embryos, we focused on 12 high-activity kinases (Supplementary Table S28). Notably, 5 and 1 pS sites in the kinase MAST3 were found in worker and drone embryos, respectively, indicating that they are hyper-phosphorylated; 2 pT in MARK2 were found in worker embryos but none in drone embryos.

Among the 1254 phosphoproteins, 84 were differentially regulated, of which 15 and 69 were up-regulated in worker and drone, respectively. The up-regulated phosphoproteins in the drone were enriched in the functional categories: cytoskeleton organization, cell death and translation elongation (Supplementary Figure S10A, Supplementary Table S29), pathways of MAPK signaling, and RNA degradation.

### Phosphoproteomic difference between the worker and drone embryos at 48 h

**FIGURE 5:**
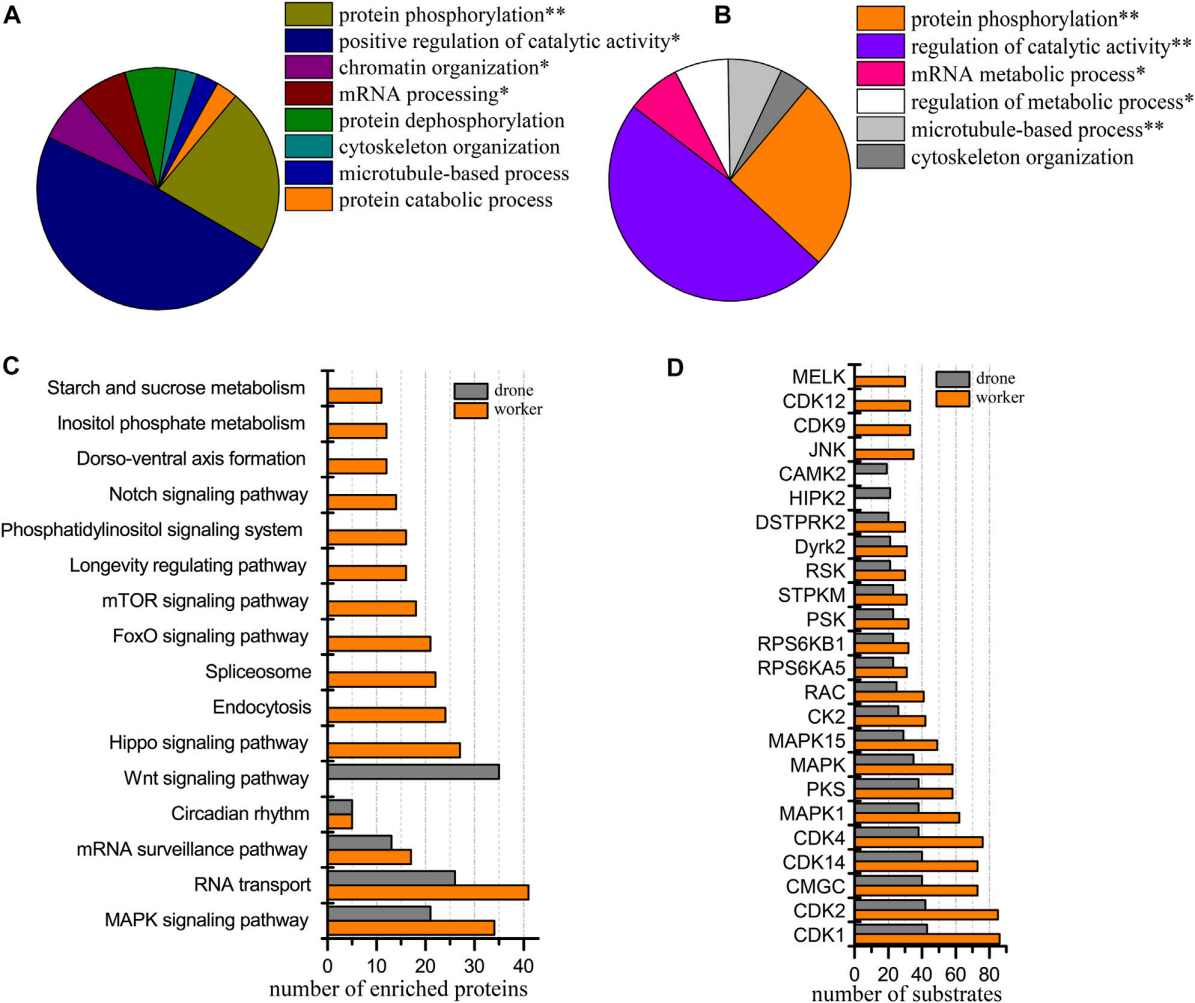
Comparison of the phosphoproteins identified in worker and drone embryos at 48 h. **(A)** ClueGO analysis of the phosphoproteins identified in 48 h worker embryos. **(B)** ClueGO analysis of the phosphoproteins identified in 48 h drone embryos. **(C)** Comparison of the biological pathways enriched in worker and drone embryo at 48 h. **(D)** Comparison of the kinase activity between the embryogenesis of worker and drone at 48 h.

At the 48 h, 3919 p-sites from 3785 phosphopeptides in 1575 phosphoproteins were identified (3601 p-sites from 3273 phosphopeptides in 1350 phosphoproteins of worker embryos and 2260 p-sites from 1973 phosphopeptides in 971 phosphoproteins of drone embryos). Phosphoproteins in worker embryos belonged to the following functional groups: protein phosphorylation, positive regulation of catalytic activity, chromatin organization, and mRNA processing (Figure 5A). In drone embryos, phosphoproteins were mainly involved in protein phosphorylation, regulation of catalytic activity, mRNA metabolic process, regulation of the metabolic process, and microtubule-based process (Figure 5B).

Both castes enriched and shared when comparing the biological pathways between the worker and drone embryos, MAPK signaling pathway, RNA transport, mRNA surveillance pathway, and circadian rhythm (Figure 5C; Supplementary Table S10). Pathways of hippo signaling, endocytosis, spliceosome, foxo signaling, mTOR signaling, longevity regulating, phosphatidylinositol, notch signaling, dorso-ventral axis formation, inositol phosphate metabolism, and starch and sucrose metabolism were uniquely enriched in worker embryos. The wnt signaling pathway was exclusively enriched in drone embryos.

Additionally, 18 kinases with high activity were compared in both embryos of worker and drone bees (Figure 5D). Kinases JNK, CDK9, CDK12, and MELK were highly activated in worker embryos, while HIPK2 and STPK were uniquely enriched in drone embryos.

Thirteen high-activity kinases were selected to compare the difference in site occupancy between worker and drone embryos (Supplementary Table S30). The kinases in worker embryos were hyper-phosphorylated. For instance, 5 and 2 pS sites in the kinase MAST3 were detected in worker and drone, respectively; 6 and 3 pS sites in the kinase Raf were observed in worker and drone, respectively.

Among the 1575 phosphoproteins in both castes, 75 phosphoproteins altered their expression levels, of which 6 and 69 were up-regulated in worker and drone, respectively (Supplementary Table S31). The up-regulated phosphoproteins in drone embryos were significantly enriched in the functional categories related to cytoskeleton organization, while no significant category was detected in worker embryos (Supplementary Figure S10B).

### Phosphoproteomic difference between the embryos of worker and drone at 72 h

**FIGURE 6:**
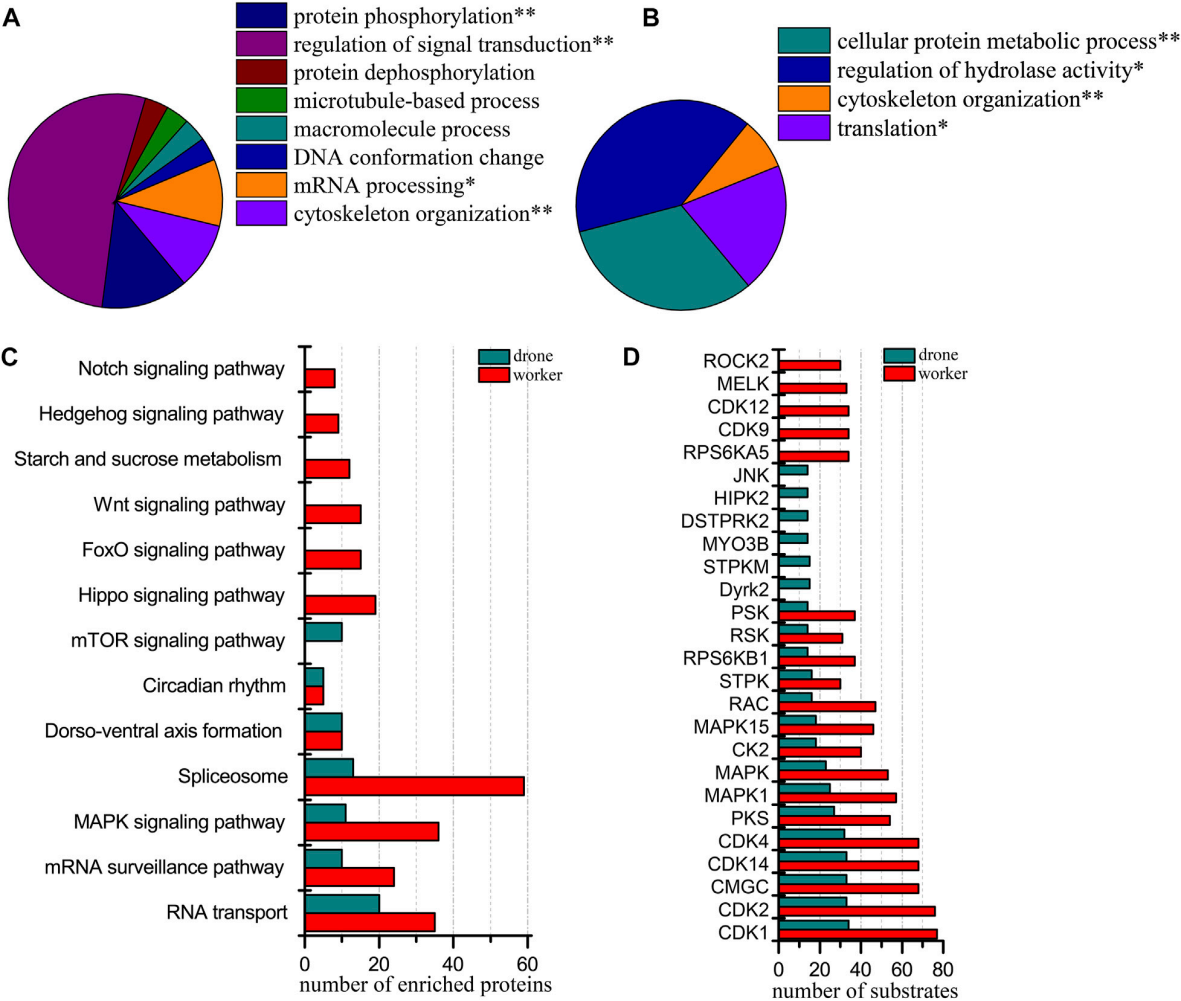
Comparison of the phosphoproteins identified in worker and drone embryos at 72 h. **(A)** ClueGO analysis of the phosphoproteins identified in 72 h worker embryos. **(B)** ClueGO analysis of the phosphoproteins identified in 72 h drone embryos. **(C)** Comparison of the biological pathways enriched in worker and drone embryo at 72 h. **(D)** Comparison of the kinase activity between the embryogenesis of worker and drone at 72 h.

At 72 h of age, 3933 p-sites from 3815 phosphopeptides in 1765 phosphoproteins were identified (3780 p-sites from 3368 phosphopeptides in 1457 phosphoproteins of worker embryos and 1622 p-sites from 1509 phosphopeptides in 836 phosphoproteins of drone embryos). Proteins in worker embryos were involved in protein phosphorylation, regulation of signal transduction, mRNA processing, and cytoskeleton organization (Figure 6A). In drone embryos, proteins were involved in the cellular protein metabolic process, regulation of hydrolase activity, cytoskeleton organization, and translation (Figure 6B).

In comparing the pathway coverage between worker and drone embryos, pathways of RNA transport, mRNA surveillance pathway, MAPK signaling, spliceosome, dorso-ventral axis formation, and circadian rhythm were enriched and shared in both castes (Figure 6C; Supplementary Table S10). Moreover, pathways of hippo signaling, foxo signaling, wnt signaling, starch and sucrose metabolism, hedgehog signaling, and notch signaling were uniquely enriched in worker embryos. The mTOR signaling pathway was exclusively enriched in drone embryos.

Furthermore, 15 kinases with high activity were found in both worker and drone embryos (Figure 6D). Kinases RPS6KA5, CDK9, CDK12, MELK, and ROCK2, were specific in worker embryos; kinases Dyrk2, STPKM, MYO3B, DSTPRK2, HIPK2, and JNK were unique in drone embryos.

Additionally, the site occupancy of 11 high-activity kinases was compared between worker and drone embryos (Supplementary Table S32). Generally, worker embryos’ kinases were more hyper-phosphorylated than in drone embryos. For example, 4 and 1 pS sites in the kinase STK10 were detected in worker and drone embryos, respectively; 17 and 12 pS sites in the kinase CDK12 were observed in worker and drone embryos, respectively.

Of the 1765 phosphoproteins expressed by worker and drone embryos at 72 h, 151 were changed in their abundance level (Supplementary Table S33). Among these, 148 and 3 were up-regulated in worker and drone, respectively. The up-regulated phosphoproteins in worker embryos were significantly enriched in DNA conformation change, organelle organization, and cytoskeleton organization (Supplementary Figure S10C), pathways of hippo signaling pathway, and spliceosome. However, no pathways or functional groups were enriched by up-regulated proteins in drone embryos.

### Verification of p-site abundance

**FIGURE 7:**
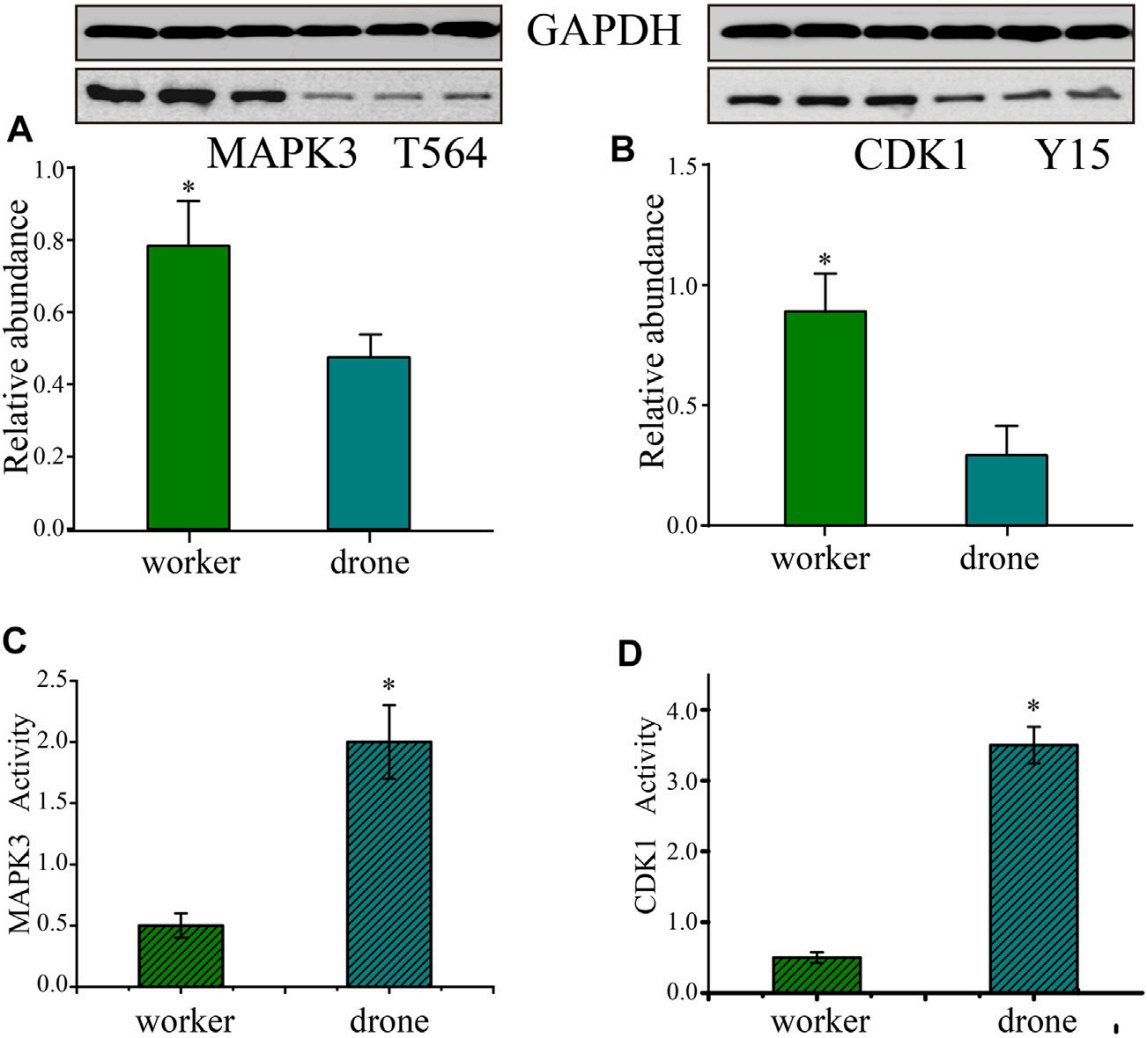
Validation of the kinases - Mitogen-activated protein kinase 3 (MAPK3) and Cyclin-dependent kinase 1 (CDK1). **(A)** Western Blotting analysis of the Thr^564^ site in MAPK3 in the embryogenesis of worker and drone at 24 h. GAPDH normalized the relative expression values of the target peptide in the two bees. **(B)** Western Blotting analysis of the Tyr^15^ in CDK1 in embryogenesis of worker and drone at 48 h. **(C)** The enzymatic activity of MAPK3 in embryogenesis of worker and drone at 24 h. **(D)** The enzymatic activity of CDK1 in embryogenesis of worker and drone at 48 h. The asterisk indicates a significant difference at *p* < 0.05.

To validate the p-site abundance differences of the crucial kinases between the embryos of worker and drone bees, two p-sites in 2 kinases were selected for Western Blotting analysis (Figures 7A,B). The abundance level of Thr^564^ in MAPK3 was greater in worker embryos at 24 h. The site abundance of Tyr^15^ in CDK1 was higher in worker embryos at 48 h.

Furthermore, the activity of CDK5 was higher in 48 h worker and drone embryos; enzymatic activity of MAPK3 was higher in drone than worker at 24 h, as well as the activity of CDK1 in 48 h embryos (Figures 7C,D).

### RNA interference

**FIGURE 8:**
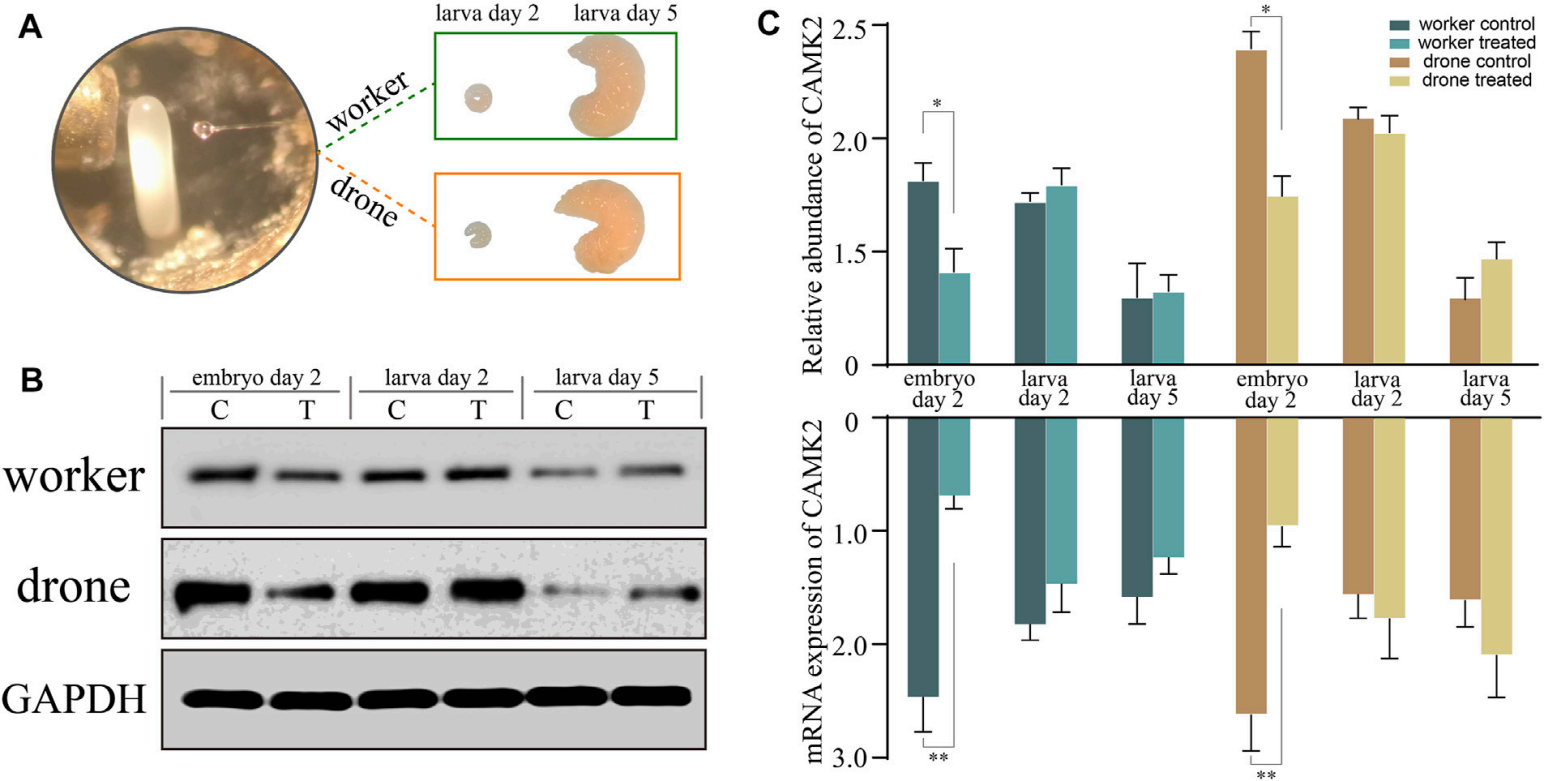
RNA interference analysis of key kinase calcium/calmodulin-dependent protein kinase II (CAMK2). **(A)** The newly laid embryos of worker and drone are subjected to gene knockdown and incubated to larvae. **(B)** The Western-Blotting bands of CAMK2 and GAPDH. **(C)** The relative expression values of CAMK2 protein and mRNA in the worker and drone at the three stages are normalized by GAPDH. The error bar is the standard deviation. The asterisks show significant differences (*p* < 0.05).

To verify the function of CAMK2 on the regulation of embryogenesis, RNA interference was tested, simulating a honey bee mutant lacking functional CAMK2. The abundance of the target decreased significantly on the following day, both in haploid and diploid embryos (Figure 8), indicating that the siRNA worked and blocked the expression of CAMK2. However, the expression recovered gradually after larva hatching due to the special effect feature. Furthermore, a similar tendency occurred in drone and worker embryos (Figure 8). Morphologically, the modified worker larvae looked right compared to the normal ones, but the drone larvae developed smaller than the ordinary. More importantly, the rapid growth of haploid larvae ceased at the 5d larval phase and could not process to the next developmental stage, while the diploid ones had no evident change in appearance.

## Discussion

To explore the regulatory mechanisms during honey bee embryogenesis, we reported the hitherto most in-depth coverage of phosphoproteome and kinome at time-resolved resolution. There were 6577 p-sites in 6342 phosphopeptides from 2438 phosphoproteins and 168 kinases. In general, embryos across the whole developmental process of both worker and drone bees employ similar phosphoproteome arsenals to regulate embryogenesis. However, distinct phosphoproteome signatures at different phases of embryos have developed in the worker and drone bees to reinforce embryogenesis.

### Worker and drone bees evolved similar phosphoproteome programs to regulate embryogenesis

The honey bee embryogenesis is physiologically distinct at different stages: division of the embryo cell and formation of the blastoderm (0–26 h), shaping of the germ band and germ layer (26–46 h), and organogenesis (46–72 h). This is in line with specific phosphoproteome signatures that have evolved to match the age-specific physiology (Fang et al., 2014). At 24 h, the uniquely induced kinases of the AGC/PKA family (PRKAC, PRKX, and PRKACA) in worker and drone are involved in broad spectra of cellular processes through phosphorylation of different nuclear and cytoplasmic substrates, such as synaptic transmission, ion channel activity, growth, and development (Feliciello et al., 2005). This finding is thought to underline the physiology of the 24 h embryos, i.e., yolk protein hydrolysis, mitosis, and blastoderm forming (Zou, 2004; Vasiev et al., 2010; Mottier-Pavie et al., 2016; Yamazaki et al., 2016). Although there are different pathways employed in worker and drone embryos at 48 h, they have similar roles in the formation of germ bands and germ layers. For instance, the mTOR signaling pathway in worker embryos may initiate or promote protein synthesis *via* PI3K/AKT or Ras/ERK signaling pathways, contributing to the accumulation of raw organic material (Lucchesi, 2004). Moreover, the regulation of protein metabolic processes enriched by the up-regulated protein may act similarly. The inositol phosphate metabolism and phosphatidylinositol signaling system, involved in intracellular signal transducing (Essen et al., 1997; Ellis et al., 1998), are likely to support the information exchange of microstructure formation. The longevity regulation pathway is inductive in protecting embryos from oxygen free radicals produced in the metabolic process (Toth et al., 2008; Donato et al., 2017). In 48 h drone embryos, the wnt signaling pathway is thought to underline the embryonic events of histogenesis. The high-activity CMGC/DYRK family kinases in 48 h embryos of worker and drone, such as Dyrk2, DSTPRK2, HIPK2, and DSTPRK2 that are vital to activation of wnt signaling pathway, play essential roles in driving the development of muscles, heart, compound eye, and brain cells (Lee et al., 2009a; Lee et al., 2009b; Elkins et al., 2009).

During embryogenesis, the organ system begins to form after 46 h. To this effect, the vast repertoire of pathways and kinases at this age in both castes suggests their critical functions in priming organogenesis. For example, the mRNA surveillance pathway can efficiently maintain protein synthesis in translational machinery (Amrani et al., 2004; Moore, 2005). Dorso-ventral axis formation is necessary for the configuration of the embryonic outline and the process of dorsal closure (Moussian and Roth, 2005; O'Connor et al., 2006) (Figure 1, Supplementary Figure S7). To this end, the pathways of notch, hippo, and wnt signaling in worker embryos are vital to promoting tissue and organ formation, thereby consolidating the nervous system configuration, mesoderm differentiation, and body segmentation (Austin and Kimble, 1987; Conlon et al., 1995; Feller et al., 2008; Hilman and Gat, 2011; Qin et al., 2013). The specifically enriched mTOR signaling pathway in drone embryos likely stimulates fatty acid metabolism and protein synthesis to hydrolyze fats and to improve the organ rudiment (Lucchesi, 2004) (Supplementary Figure S7). Moreover, the unique high-activity kinase JNK (MAPK family) and STE20 family kinase MYO3B in drone embryos may relate to the epithelial healing and motility of molecular motor myosin, conducing to the process of dorsal closure (Riesgo-Escovar and Hafen, 1997; Wu et al., 1997) (Supplementary Figure S8). Furthermore, phosphorylation events are also involved in the p-site-mediated modulation. For example, the hyper-phosphorylated CDK kinases, CDK2, CDK1, and CDK12 in worker embryos suggest that kinase activity is induced to catalyze the target proteins, thus driving the cell cycle to cement the needs of organogenesis (Supplementary Table S14). As a negative indicator of kinase Raf (Moelling et al., 1984; Morrison and Cutler, 1997; Zimmermann and Moelling, 1999) in drone embryos (Supplementary Table S26), the dephosphorylation of Ser^259^ manifests the fact that the kinase activity is enhanced, which is critical to stimulating cell growth, differentiation, and apoptosis during embryogenesis (Roskoski, 2010). Taken altogether, the worker and drone bees may develop a shared central regulatory mechanism to prime embryogenesis and evolve unique phosphorylation events to match age-specific physiology. These findings are in concordance with the fact that a common and age-related proteome is necessary to prime embryonic development in worker and drone bees (Fang et al., 2015).

### Different phosphoproteomes evolved at different ages between worker and drone embryos

Although 24 h embryos are faced with the same tasks of producing energy, promoting mitosis, and initializing the configuration of embryonic tissues, the worker and drone embryos differ in kinase-modulated pathways. For instance, three pathways specifically enriched in worker embryos (starch and sucrose metabolism, endocytosis, and spliceosome) (Figure 4C) indicate that the embryo cells initiate the conversion of stored nutrients from yolk into metabolic energy through endocytosis as the fuel to stimulate cell division and gene transcription. Moreover, the enriched microtubule-based process and positive regulation of catalytic activity in worker embryos (Figure 4A) suggest that the critical biological events are to produce metabolic energy and launch cell mitosis. Furthermore, as the negative regulatory factor of cell cycle, the uniquely activated kinase STPKM (serine/threonine-protein kinase minibrain, mnb) at 24 h could induce the expression of protein Dacapo, which is an inhibitor to CDKs (Shaikh et al., 2016). The expressed mnb indicates that the activities of mitotic proliferation, cell cycle, and blastoderm formation are restricted to some extent. For the drone embryos, the uniquely enriched pathway RNA transport (Figure 4C) suggests the significance of translational machinery for protein synthesis to underpin organ construction and organization. Moreover, the MAPK signaling pathway and RNA degradation pathway are enriched by the up-regulated proteins in drone embryos (Figure 4C). This illustrates their enhanced roles in driving gene expression, cell proliferation, differentiation, and migration for the upcoming formation of the blastoderm and germ band. Furthermore, phosphorylation by CDK-activating kinase on the T-loop region is a prerequisite for activating kinase cyclin-dependent kinase 12 CDK12 (CDK12) (Bösken et al., 2014). The higher number of observed p-sites on CDK12 in drone embryos (Supplementary Table S26) explains the induced function of promoting gene transcription and RNA maturity in drones (Bartkowiak et al., 2010). The Thr^564^ on MAP/microtubule affinity-regulating kinase 3 (MAPK3) catalyzed by PRKCZ/aPKC harms kinase activity (Hurov et al., 2004; Lizcano et al., 2004). The observed pT on site 564 in MAPK3 in worker embryos (Supplementary Table S28), validated by Western Blotting analysis (Figure 7A), indicates that the activity of MAPK3 is inhibited (verified by kinase activity assay) (Figure 7C), thus leading to certain inactivated biological events, such as gene transcription and cytoskeleton building. The N-terminal phosphorylation of microtubule-associated serine/threonine-protein kinase 3 (MAST3) is the precondition for ubiquitination, which triggers proteasome-mediated MAST3 degradation (Xiong et al., 2004; Zhou et al., 2004). The hyper-phosphorylated MAST3 in worker embryos (Supplementary Table S28), relative to in drone embryos, could explain the higher activity of MAST3 in drone embryos, which is vital in intracellular signal transduction and cytoskeleton organization (Lumeng et al., 1999; Zhou et al., 2004). These kinases described above are thought to be the main reason for the significantly enriched protein phosphorylation in drone embryos (Figure 4B). Considering these findings, the main regulatory events in 24 h worker embryos promote cell proliferation, whereas, in drone embryos, they underscore protein synthesis for tissue block and the cytoskeleton.

The primary process in the middle stage of honey bee embryos is the establishment of embryonic tissues and organs. The worker and drone, however, resort to different pathways and kinases to achieve this embryonic development. Here, in worker bees, the uniquely enriched 11 pathways (Figure 5; Supplementary Table S10) indicate that phosphorylation is involved in comprehensive scenarios of biochemical routes to consolidate embryogenesis by providing metabolic energy and protein molecules for the construction of embryonic tissues and organs. Examples of such pathways are notch signaling, hippo signaling, and dorsal-ventral axis formation (Austin and Kimble, 1987; Conlon et al., 1995; Moussian and Roth, 2005; O'Connor et al., 2006; Feller et al., 2008; Hilman and Gat, 2011; Qin et al., 2013). The specifically enriched wnt signaling pathway and cytoskeleton organization in drone embryos (Figure 5C) indicate that the molecular events involved in the formation of tissues and organs are predominant at this stage (Zou, 2004; Vasiev et al., 2010; Mottier-Pavie et al., 2016; Yamazaki et al., 2016). Furthermore, the highly activated kinase calcium/calmodulin-dependent protein kinase II (CAMK2) was functionally investigated by gene knockdown (Figure 8). CAMK2 participates in WNT/Ca^2+^ signaling pathways, regulating developmental processes such as cytoskeletal rearrangements, dorsoventral patterning, and embryo tissue separation (Kohn and Moon, 2005). The RNAi-treated drones were more dependent on CAMK2 during the early stage of embryogenesis (Figure 8), probably because of the earlier occurrence of morphogenesis in the drone than worker (Fang et al., 2015); the knockdown of CAMK2 in the embryo may perturb dorsal cell fate (Garriock and Krieg, 2007) and have a substantial impact on the development process of drone embryo. In worker embryos, the high-activity kinase cyclin-dependent kinase (CDK) 12 (Supplementary Table S14) suggests the continuously high activity of RNA transcription (Bartkowiak et al., 2010) to promote the maturity of biological materials of tissue and organ. CDK1 regulates the centrosome cycle and the initiation of mitosis and promotes the G2 to M phase transition, thus regulating the cell cycle. The phosphorylation of Thr^15^ in CDK1 in worker embryos (Supplementary Table S30), induced to inactivate itself (Meijer et al., 1997; Hu et al., 2007), suggests the activity related to the cell cycle in worker embryos is attenuated. This is validated by Western Blotting analysis (Figure 7B) and kinase activity assay (Figure 7D). raf homolog serine/threonine-protein kinase Raf (Raf) is critical for forming the anterior-posterior axis and developing gastrulation and imaginal disc (Li et al., 2003; Pallavi and Shashidhara, 2003). The highly phosphorylated Raf restrains the activation of signal pathways related to transmitting information from the extracytoplasmic space to the nuclei during the embryogenesis of *Drosophila* (Sprenger et al., 1993). The observed hyper-phosphorylation on Raf in worker embryos (Supplementary Table S30) illustrates that the tissue organization activity in worker embryos is weaker than in drone embryos. Like in 24 h embryos, hyper-phosphorylated MAST2 in worker embryos weakens the activity involved in intercellular signal transduction and cytoskeleton organization (Supplementary Table S30). Although the worker embryos launch a series of pathways associated with embryonic construction, many negative modulators, such as the essential kinases, may attenuate their activity for rudiment organization compared to drones. Hence, for the 48 h embryos, the phosphorylation in workers may underpin organ organization, whereas, in drone embryos, the phosphorylation seems to establish organs.

The last phase of embryogenesis mainly configures the tissues and organs, which is different from the previous stages. As expected, the enriched pathways in worker embryos will likely serve this exact purpose (Figure 6C). Examples of such pathways are hippo signaling, wnt signaling, and hedgehog signaling (Mohler, 1988; Zou, 2004; Vasiev et al., 2010; Mottier-Pavie et al., 2016; Yamazaki et al., 2016). The mTOR signaling pathway-specific in drone embryos (Figure 6C) could be explained by its role in the degradation of fatty acids, which is critical to providing both metabolic energies and forming the stomach and intestines (Fleig and Sander, 1986b). Also, the pathways of hippo signaling and spliceosome activated by up-regulated phosphoproteins manifest their functions to prime protein synthesis and organogenesis. The induced kinases in worker embryos, CDK9, CDK12, and mnb (Figure 6D), are suggestive of their importance in regulating the processes of cell growth and muscle differentiation (Grana et al., 1994; Simone et al., 2002) and the formation of optical nerves in the late stage of embryonic development (Tejedor et al., 1995) as found in *Drosophila*. These findings signify that the worker embryo is approaching a stage of massive tissue differentiation and organ formation. However, the highly-induced kinase MYO3B found in drone embryos (Figure 6D) is closely related to the organization of actin cytoskeleton (Pruyne and Bretscher, 2000; Galletta et al., 2008). This indicates that embryonic improvements are still ongoing, such as blastokinesis and dorsal closure. STK10 is essential in microtubule organization, cell apoptosis, and migration (Quizi et al., 2013). Its degree of autophosphorylation is proportional to its activity (Yamada et al., 2000). The higher number of p-sites on SLK in worker embryos (Supplementary Table S32) suggests that the activity of SLK is enhanced in worker embryos. Moreover, the higher number of p-sites on CDK12 of worker embryos (Supplementary Table S32) implies that it is activated, and thus the transcriptional and translational machinery is higher than in drone embryos (Bartkowiak et al., 2010). The phosphorylation of Ser^637^ and Ser^628^ of AbI (tyrosine-protein kinase, Abl) is negative to the kinase activity (Jung et al., 2008). The observed Ser^637^ in Abl of drone embryos (Supplementary Table S32) may reduce the kinase activity, suggesting that the biological processes in which Abl is involved are functionally enhanced, such as cytoskeleton organization, epidermal neural differentiation, and cell cycle regulation (Grevengoed et al., 2001). The activation of these kinases may be the reason for the significantly enriched functional category of protein phosphorylation in worker embryos (Figure 6A). In brief, phosphorylation in the late stage of worker embryos may facilitate tissue differentiation and organ configuration. In contrast, it may improve embryonic rudiment, blastokinesis, and dorsal closure in drone embryos.

## Conclusion

This study provides a comprehensive resource that reveals phosphorylation-mediated regulatory mechanisms during honey bee embryogenesis. The identified >6000 p-sites, >140 kinases, and the subcellular and chromosome information are the first reported to reveal the phosphorylation networks in honey bee embryos. This offers a valuable resource for signaling research on regulatory sites, kinases, and dynamics in honey bee embryos. Further study is encouraged to understand the role of regulated p-sites and kinases during honey bee embryogenesis.

## Data Availability

The datasets presented in this study can be found in online repositories. The names of the repository/repositories and accession number(s) can be found below: The mass spectrometry proteomics data have been deposited to the ProteomeXchange Consortium (http://proteomecentral.proteomexchange.org) via the iProX partner repository (Ma et al., 2019) with the dataset identifier PXD033311.
