# Supplementary figures and images for: Revealing phosphorylation regulatory networks during embryogenesis of honey bee worker and drone (Apis mellifera)
Source: Front Cell Dev Biol. 2022 Sep 26;10:1006964. doi: 10.3389/fcell.2022.1006964 (PMC9548569; doi:10.3389/fcell.2022.1006964)

Figure S1

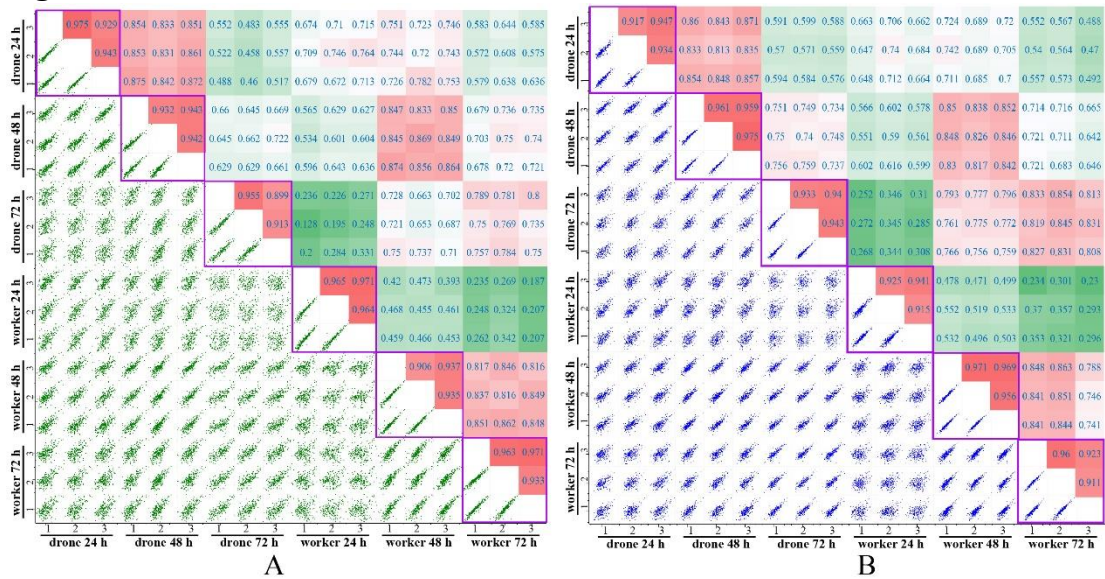

Figure S2

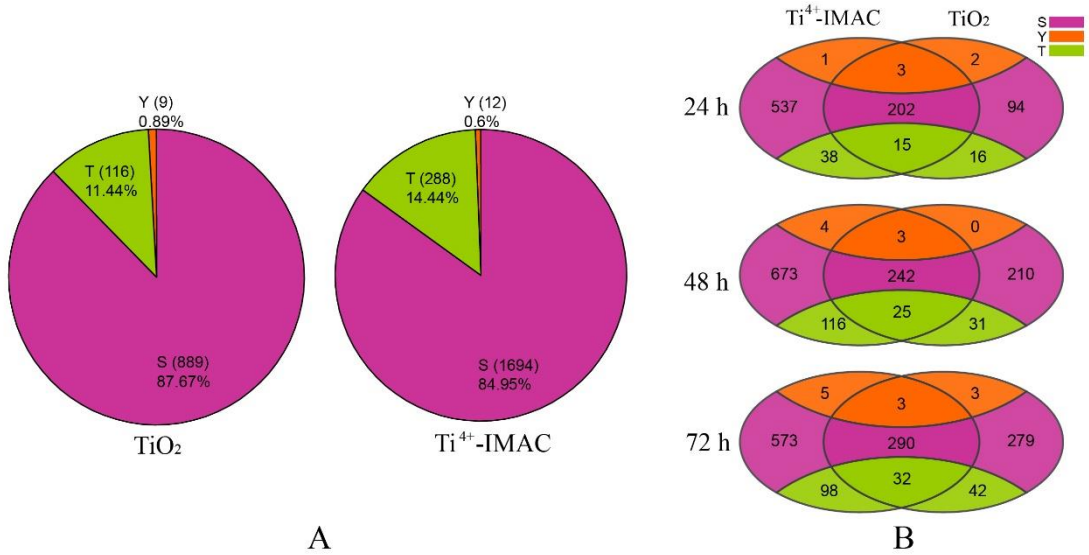

Figure S3

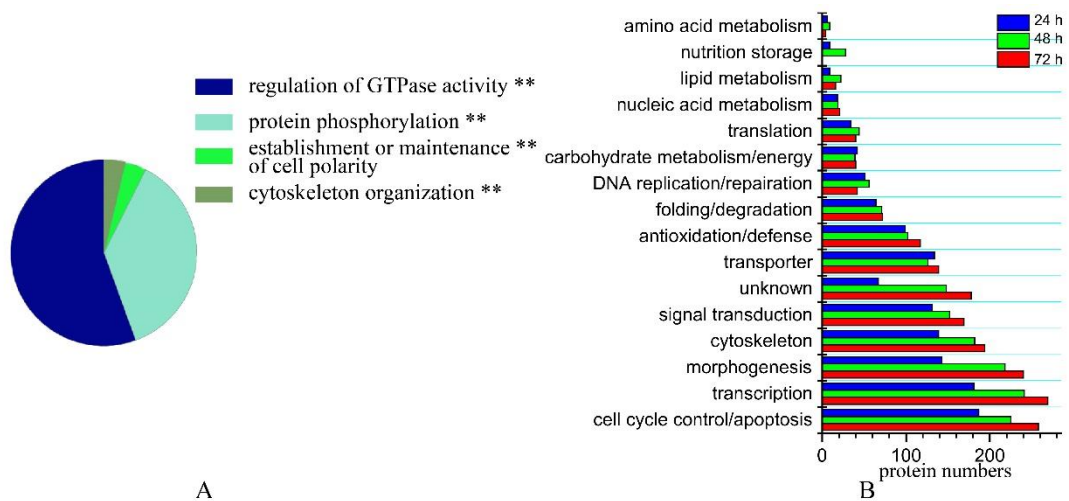

**Figure S4**

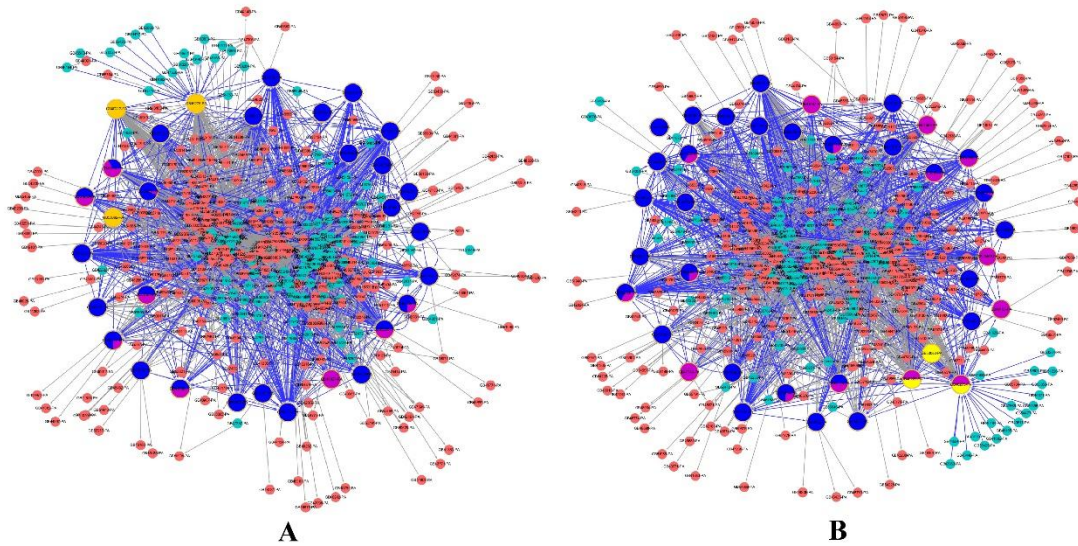

**Figure S5**

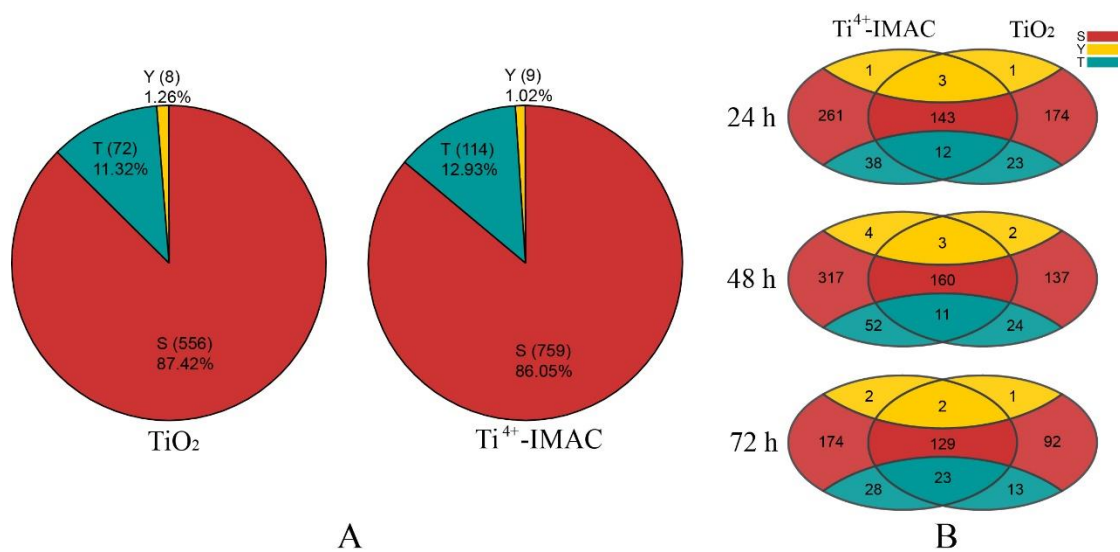

Figure S6

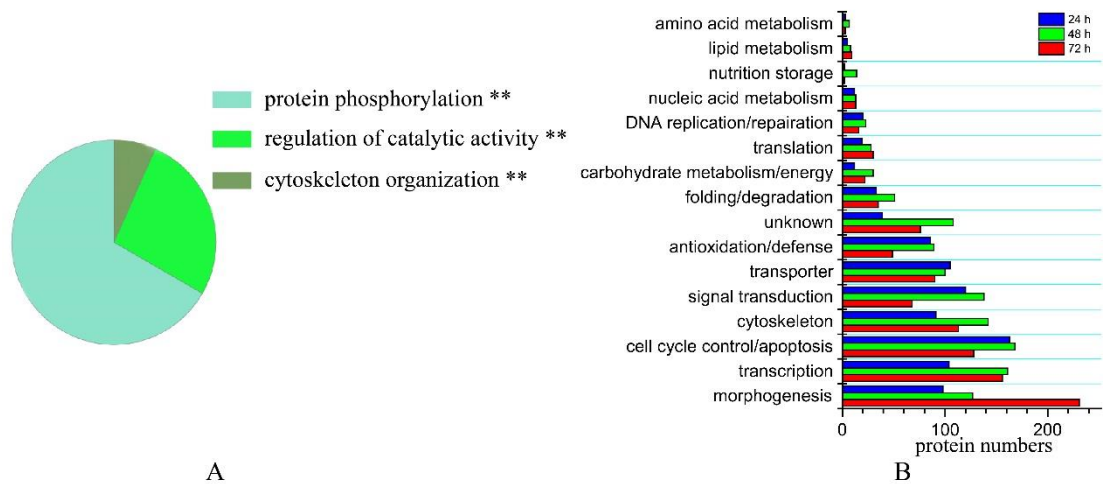

Figure S7

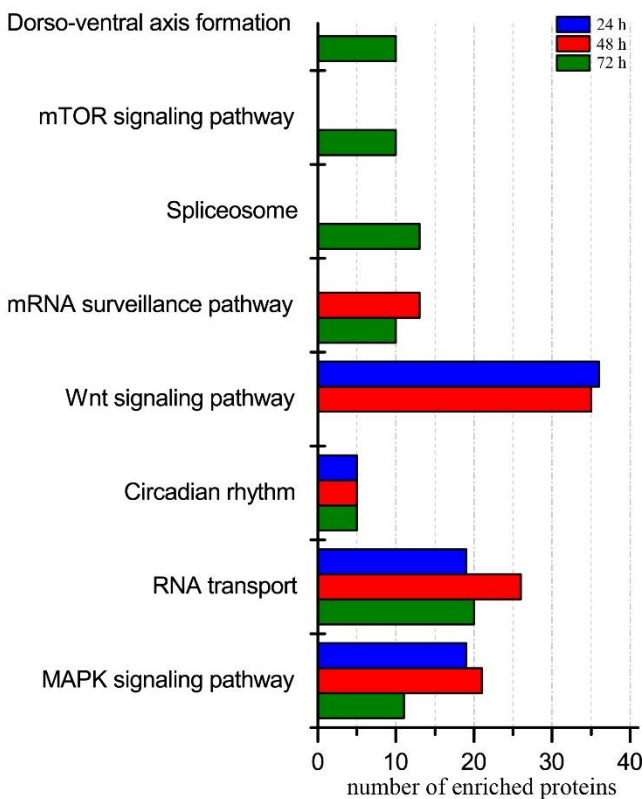

Figure S8

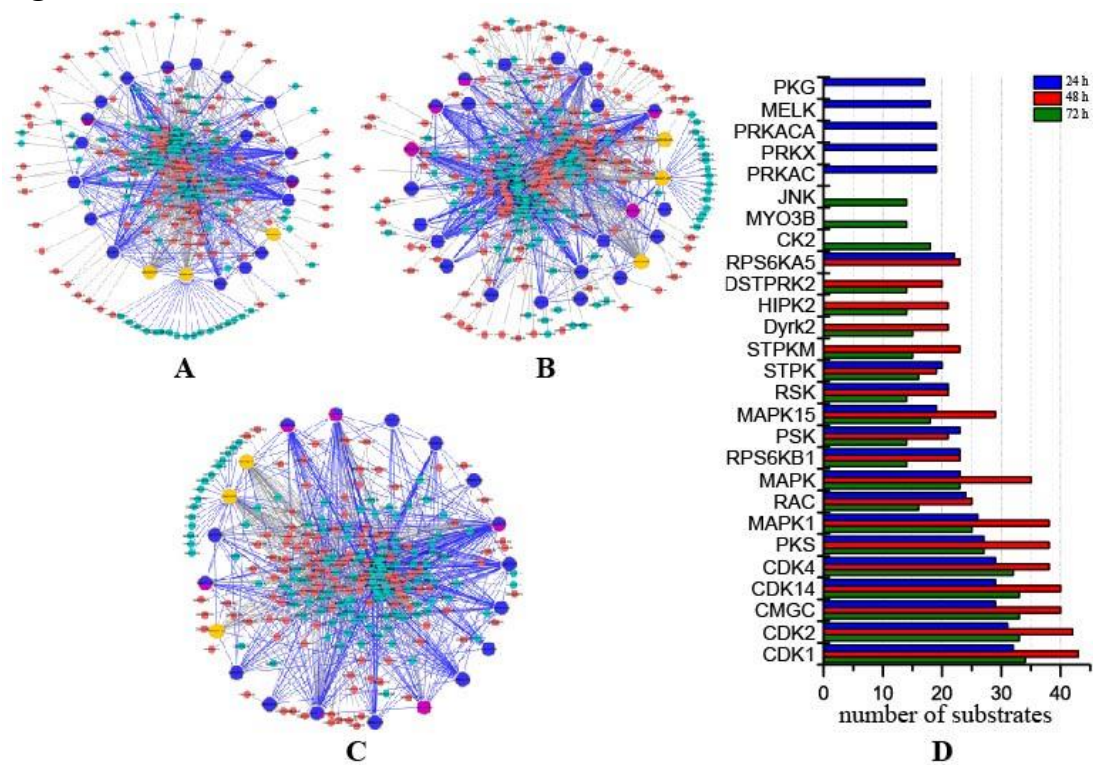

Figure S9

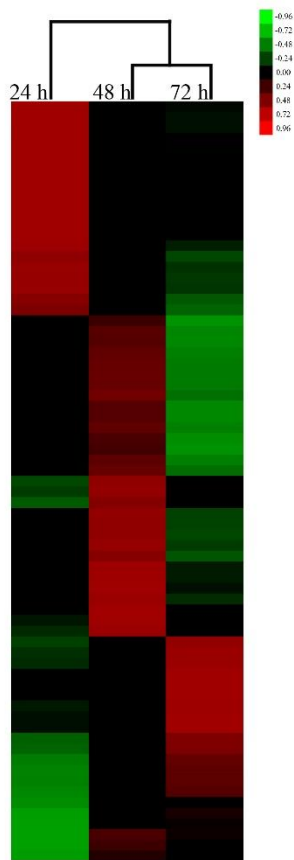

**Figure S10**

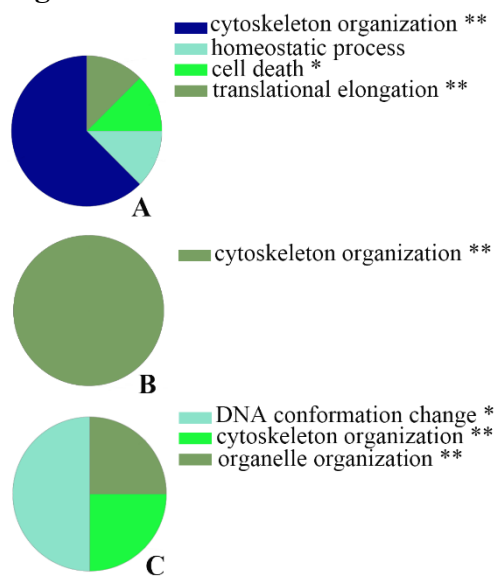

Supplement: Supplementary file 1 [file DataSheet1.PDF]
